# Comorbidities of chronic rhinosinusitis in children and adults

**DOI:** 10.1002/clt2.12354

**Published:** 2024-04-24

**Authors:** Aada Murtomäki, Alma Helevä, Paulus Torkki, Jari Haukka, Anna Julkunen‐Iivari, Riikka Lemmetyinen, Mika Mäkelä, Aarno Dietz, Mikko Nuutinen, Sanna Toppila‐Salmi

**Affiliations:** ^1^ Inflammation Center Skin and Allergy Hospital Helsinki University Hospital and University of Helsinki Helsinki Finland; ^2^ Department of Otorhinolaryngology University of Eastern Finland Jouensuu, Kuopio Finland; ^3^ Programme of the Faculty of Medicine University of Helsinki Helsinki Finland; ^4^ Department of Public Health University of Helsinki Helsinki Finland; ^5^ Department of Otorhinolaryngology Kuopio University Hospital Wellbeing Services County of North Savo Kuopio Finland

**Keywords:** allergy, asthma, chronic rhinosinusitis, non‐steroidal anti‐inflammatory drug exacerbated respiratory disease, rhinitis

## Abstract

**Background:**

Chronic rhinosinusitis (CRS) is a chronic inflammatory disease of the nose and paranasal sinuses lasting ≥12 weeks. CRS may exist with (CRSwNP) or without (CRSsNP) nasal polyps. The aim was to evaluate conditions associated with CRS in a randomized hospital cohort. We hypothesized that comorbidities and surgical procedures differ between pediatric and adult patients.

**Methods:**

This study consisted of hospital registry data of a random sample of rhinosinusitis patients (age range 0–89 years) with the diagnosis of J32 or J33, correspondingly, registered during outpatient visits from 2005 to 2019 (*n* = 1461). The covariates of interest were collected from electronic health records based on ICD‐10 codes and keyword searches.

**Results:**

Among pediatric patients (*n* = 104), the relative proportions of CRSsNP and CRSwNP were 86% and 14% respectively. The relative proportions of adult patients (*n* = 1357) with CRSsNP and CRSwNP were 60% and 40%, respectively. The following comorbidities significantly differed (*p* < 0.05) between pediatric and adult populations: allergy, chronic otitis media, and tonsillar diseases. In total, 41 % of the children and 46% of the adults underwent baseline endoscopic sinus surgery (ESS). Additional surgeries of the ear, nose and pharynx were significantly more common among children compared with adults. Risk of revision after baseline ESS was associated (*p* < 0.05) with allergy, asthma, eosinophilia, CRSwNP, immunodeficiency or its suspicion, non‐steroidal anti‐inflammatory drug exacerbated respiratory disease, and number of any diseases ≥2.

**Conclusions:**

Our study showed that comorbidities differ between pediatric and adult rhinosinusitis patients, as allergy, asthma and allergy, chronic otitis media, mental health disorders, and tonsils disease were significantly more prevalent among pediatric patients. Children and adults were equally treated with ESS. Notably, children underwent additional surgery on adenoids and tonsils more frequently. The effectiveness of ESS in multimorbid adults should be assessed at an individual level.

## 1 BACKGROUND

Chronic rhinosinusitis (CRS) is a symptomatic inflammatory disease of the nasal and paranasal mucosa lasting more than 12 weeks. Based on the appearance of the nasal cavities on endoscopy, CRS can be classified into two subforms: with nasal polyps (CRSwNP) and without (CRSsNP). These phenotypes are thought to have different etiologies, pathomechanisms and degrees of inflammation.^1^ The prevalence of CRS in Europe is estimated to be 11%,^2^ and that of CRSwNP is 1%–4%.^1^ Pediatric CRS (PCRS) affects 2.1%–4% of the population.

CRS and PCRS patients are likely to present with asthma.^1^, ^3^ Other CRS risk factors include anti‐inflammatory drug‐exacerbated respiratory disease (NERD),^1^ dose‐dependent correlation of firsthand and secondhand smoking,^4^ genetic predisposition,^5^ microbes,^6^ and pollutants.^7^ In addition, variations in sinonasal anatomy appear to affect CRS progression.^1^

Differences in histopathological pathogenesis in pediatric and adult forms of CRS have been acknowledged.^8^, ^9^ However, information on adult CRS is more well‐documented compared with that on children. Although clinical differences between pediatric and adult CRS can be noted in hospital settings, there is limited literature on the subject. Children with CRS commonly experience cough and rhinorrhea,^10^ along with other non‐specific and subtle symptoms,^1^ whereas adults experience hyposmia, postnasal discharge and nasal obstruction.^11^ Because of difficulties in tolerating nasal endoscopy and the limitations of sinus radiography in uncooperative children, clinical symptoms are often the primary means of diagnosis.

A surgical approach is considered in patients who are refractory to conservative treatment. Endoscopic sinus surgery (ESS) is the most common approach in pediatric and adult patients; however, adenoidectomy is often recommended to prevent the need for ESS in children.^1^ ESS usually has a high initial success rate of 76%–98%.^12^ CRSwNP patients have more frequent disease recurrence than CRSsNP patients, yet both groups have been shown to benefit from ESS.^13^

Improved recognition and early intervention of CRS are needed to understand the factors behind the development of CRS in different age patients and prevent long‐term consequences. There is still limited knowledge of the differences between pediatric and adult CRS and putative risk factors behind revision ESS as a sign of uncontrolled disease. The aim was to evaluate pediatric and adult CRS in a hospital setting: relative proportion of different phenotypes, comorbidities, ESS, and risk factors associated with revision surgery. We hypothesized that comorbidities and surgical procedures differ between pediatric and adult patients.

## 2 METHODS

### 2.1 Study design

This is a retrospective registry‐based quantitative analysis of CRS in Finland. We analyzed the electronic health records (EHR) of 1461 pediatric and adult patients. Records were collected from 2005 to 2019.

### 2.2 Patients

This retrospective registry‐based follow‐up study was carried out on hospital registry data of a random sample of rhinosinusitis patients visiting the Departments of Otorhinolaryngology at the Hospital District of Helsinki and Uusimaa (HUS), Finland. The ethical committee of the Hospital District approved the study (no. 31/13/03/00/2015) and agreed that there was no need for written informed consent.

The initial cohort (*n* = 5080) consisted of rhinosinusitis patients diagnosed with ICD‐10 diagnosis of J30, J31, J32, J33 or J01 registered during outpatient visits in 2005, 2007, 2009, 2011 or 2013. The sample size was maintained at a consistent level for each year and month. The last data collection day of the follow‐up data was 31.9.2019. Our previous study initially presented this patient population.^14^ Here, we used subsample data of all the cohort's rhinosinusitis subjects with CRSsNP (J32) or CRSwNP (J33) diagnosis (*n* = 1461).

Collected covariates included personal characteristics (gender, age), disease of interest (CRS), phenotypes of interest (CRSwNP, CRSsNP), and coexisting diseases (see Nuutinen et al.^14^ for a detailed description of the used ICD‐10 codes and keyword searches). Diagnosis J33 was defined as CRSwNP. Diagnosis J32 without J33 and existing EHR of nasal polyps was defined as CRSsNP.

The child and adult populations were classified according to the patient's age at their initial outpatient visit. The child population was defined as individuals under 18 at their first visit.

ESS operations of the nose and maxillary antrum, as well as surgery of the ethmoidal, frontal and sphenoidal sinuses, were included in our study.^15^ Ethmoidectomy was divided into partial and total sections based on the extent of the surgery. Total ethmoidectomy was a physician‐reported opening of all ethmoidal cells ± additional operations. Ethmoidectomy was considered partial if there was no indication of the opening of all ethmoidal cells in the EHR. Baseline ESS was defined as the first sinus operation during the follow‐up period. ESS was considered a revision operation if the surgery was performed after baseline ESS during the follow‐up. Unfortunately, data on the surgeries performed before the start of the follow‐up was unavailable. Hence, patients might have undergone surgery prior to the baseline ESS.

Additional surgeries of the ear, nose and pharynx included tympanostomy, balloon catheter sinuplasty, adenoidectomy, adenotonsillectomy, and tonsillectomy.

### 2.3 Information extraction from electronic health records

The information extraction method from clinical texts is based on two separate methods that have been previously described.^14^ In brief, we first searched directly for ICD‐10 codes from the clinical texts. Secondly, we searched keywords related to basic diseases (such as “diabetes” and “NERD”).

In EHR, CRS and CRSwNP diagnoses were based on the European Position Paper on Rhinosinusitis and Nasal Polyps guidelines.^16^, ^17^, ^18^ Asthma diagnosis was based on ICD‐10 code J45. And/or a positive keyword search for “asthma.” Allergy diagnosis was based on ICD‐10 codes J30 or J45.0 and a positive keyword search for “cat,” “birch,” “prick,” “skin prick test,” or “rast.” The keywords for NERD were: “aerd,” “samter,” “aspirin,” and “asa.” Immunodeficiency was defined by ICD‐10 codes B20, D80‐D84 or a positive finding in a keyword search for “immunodeficiency” in EHR. Immunodeficiency suspicion was defined by a positive keyword search for “infectious disease doctor,” “susceptibility to infection,” or “immune deficiency.”

### 2.4 Statistical analyses

A comparison of statistical differences in comorbidities and surgical operations between child and adult patients was conducted using logistic regression analysis. Odds ratios (OR) are reported with 95% confidence intervals (CI).

**FIGURE 1:**
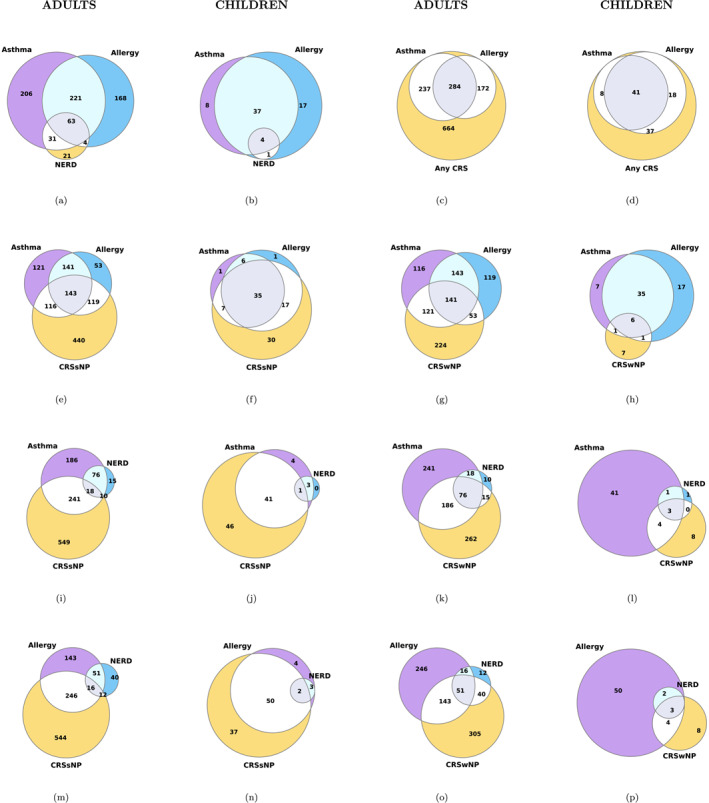
Venn diagrams illustrate the total count of pediatric and adult patients with overlapping diagnoses of chronic rhinosinusitis and closely related diseases. CRS, chronic rhinosinusitis; CRSsNP, chronic rhinosinusitis without nasal polyps; CRSwNP, chronic rhinosinusitis with nasal polyps; NERD, non‐steroidal anti‐inflammatory drug exacerbated respiratory disease. Note that NERD is not an independent disease but is associated with asthma and/or CRS.

The Cox proportional‐hazard model was used to evaluate the association between exposure factors and the need for revision ESS in adult‐type CRS. Multivariable models with minimal sufficient adjustment sets were conducted to estimate the total effect of selected exposure factors (asthma, CRSwNP and NERD) on revision ESS. The frequency‐matching variables (age and gender) were included as covariates in all analyses. A directed acyclic graph (DAG) was used to inform the choice of other covariates. The following known or possible ESS revision risk factors were included in the causal diagram: allergy, asthma, baseline total ethmoidectomy, CRSwNP, NERD and previous ESS (see Figure 1). Associations between those variables and the direction of the associations were based on the literature^1^, ^19^, ^20^ and expert knowledge. Hazard ratios are reported with 95% CI and *p*‐values. This analysis focused exclusively on adult patients because the number of PCRS patients requiring revision ESS (*n* = 2) was insufficient for inclusion.

Statistical analyses were performed using R (version 4.1.0),^21^ Survival,^22^ Dplyr,^23^ and Rcpp^24^ packages.

## 3 RESULTS

### 3.1 Comorbidities among children

Characteristics and comorbidities of our child population are shown in Table 1. Our patient population (*n* = 1357) included 104 (8%) pediatric patients, of which 59 (57%) were female. The average number of comorbidities (±SD) was 1.9 ± 1.6, and the mean follow‐up time was 8.6 ± 3.3 years. The mean age of the pediatric population was 10.4 ± 5.3 years and was defined at the time of the first visit. The mean number of visits was 6.8 ± 6.9 per follow‐up, and the average time between two visits during follow‐up was 195 ± 188 days. The relative proportions of patients with CRSsNP and CRSwNP were 86% and 14%, respectively. Patients with CRSsNP diagnosis were the youngest (10.3 ± 5.3 years) and had the highest number of visits (6.9 ± 7.3). The CRSwNP phenotype had a female minority (40%).

**TABLE 1:** Comorbidities in children.

Variable	All patients	CRSsNP	CRSwNP
Patients, *n* (%)	104 (100)	89 (85.58)	15 (14.42)
Females, *n* (%)	59 (56.73)	53 (59.55)	6 (40)
Age, mean (SD)	10.4 (5.25)	10.29 (5.26)	11.07 (5.28)
Number of comorbidities, mean (SD)	1.89 (1.62)	1.93 (1.58)	1.67 (1.88)
Number of any comorbidities 0–1, *n* (%)	54 (51.92)	47 (52.81)	7 (46.67)
Number of any comorbidities >1, *n* (%)	54 (51.92)	47 (52.81)	7 (46.67)
Number of any comorbidities >3, *n* (%)	20 (19.23)	17 (19.1)	3 (20)
Number of any comorbidities >5, *n* (%)	3 (2.88)	2 (2.25)	1 (6.67)
Number of visits, mean (SD)	6.8 (6.9)	6.9 (7.29)	6.2 (3.9)
Number of visits, pulmonology and allergy, mean (SD)	0.01 (0.1)	0.01 (0.11)	0 (0)
Number of visits, pulmonology, mean (SD)	0.53 (1.55)	0.6 (1.65)	0.13 (0.52)
Number of visits, ENT, mean (SD)	6.26 (6.85)	6.29 (7.26)	6.07 (3.83)
Frequency of visits, (visits/follow‐up, from first to last visit), mean (SD)	7.91 (20.7)	8.51 (22.21)	4.39 (5.91)
Frequency of visits, (visits/follow‐up, from first to end), mean (SD)	0.9 (0.97)	0.92 (1.02)	0.77 (0.58)
Time interval between visits (days), mean (SD)	195.23 (187.83)	202.62 (196.21)	146 (111.04)
Follow‐up time (years), mean (SD)	8.57 (3.33)	8.57 (3.46)	8.57 (2.4)
Allergy, *n* (%)	59 (56.73)	52 (58.43)	7 (46.67)
Asthma, *n* (%)	49 (47.12)	42 (47.19)	7 (46.67)
Chronic otitis media, *n* (%)	20 (19.23)	20 (22.47)	0 (0)
Diabetes, *n* (%)	10 (9.62)	9 (10.11)	1 (6.67)
Eosinophilia, *n* (%)	13 (12.5)	12 (13.48)	1 (6.67)
Immunodeficiency, *n* (%)	0 (0)	0 (0)	0 (0)
Immunodeficiency or its suspicion, *n* (%)	4 (3.85)	3 (3.37)	1 (6.67)
NERD, *n* (%)	5 (4.81)	2 (2.25)	3 (20)
Other chronic pulmonary diseases, *n* (%)	15 (14.42)	12 (13.48)	3 (20)
Tonsils disease, *n* (%)	22 (21.15)	20 (22.47)	2 (13.33)

Abbreviations: CRSsNP, chronic rhinosinusitis without nasal polyps; CRSwNP, chronic rhinosinusitis with nasal polyps; ENT, ear, nose, and throat diseases; NERD, non‐steroidal anti‐inflammatory drug exacerbated respiratory disease.

Comorbidities with the highest relative proportion were allergy (57%) and asthma (47%). The relative proportion of comorbid allergy among patients with CRSsNP and CRSwNP was 58% and 47%, respectively. The relative proportion of comorbid asthma was equal (47%) among patients with CRSsNP and CRSwNP. The relative proportions of NERD among CRSsNP and CRSwNP were 2.3% and 20%, respectively.

Tonsils disease was in 21%, chronic otitis media (COM) in 19%, other chronic pulmonary diseases in 14%, eosinophilia in 10%, diabetes in 10%, and immunodeficiency or its suspicion in 4.0% of cases as comorbidity. Our cohort had no CRS patients with cystic fibrosis (CF) or primary ciliary dyskinesia (PCD).

### 3.2 Comorbidities among adults

Characteristics and comorbidities of our adult population are shown in Table 2. In our study, the adult population consisted of 1357 patients, of which 59% were female. The mean (±SD) age for the adult population was 45.1 ± 15.3 years. The average number of comorbidities was 1.4 ± 1.4, and the mean follow‐up time was 8.6 ± 3.5 years. The mean number of visits was 8.8 ± 11.8 per follow‐up, and the average time between two visits during follow‐up was 207 ± 266 days. The relative proportions of patients with CRSsNP and CRSwNP were 60% and 40%, respectively. Patients with CRSsNP were the youngest (43.1 ± 15.2 years) and their frequency of visits during follow‐up from the first to the last visit was the greatest (8.7 ± 18.2 days). CRSwNP patients had a female minority (40%) (Table 2).

**TABLE 2:** Comorbidities in adults.

Variable	All patients	CRSsNP	CRSwNP
Patients, *n* (%)	1357 (100)	818 (60.28)	539 (39.72)
Females, *n* (%)	800 (58.95)	587 (71.76)	213 (39.52)
Age, mean (SD)	45.06 (15.26)	43.11 (15.22)	48.03 (14.86)
Number of comorbidities, mean (SD)	1.44 (1.43)	1.22 (1.34)	1.78 (1.49)
Number of any comorbidities 0–1, *n* (%)	544 (40.09)	277 (33.86)	267 (49.54)
Number of any comorbidities >1, *n* (%)	544 (40.09)	277 (33.86)	267 (49.54)
Number of any comorbidities >3, *n* (%)	143 (10.54)	57 (6.97)	86 (15.96)
Number of any comorbidities >5, *n* (%)	14 (1.03)	8 (0.98)	6 (1.11)
Number of visits, mean (SD)	8.84 (11.77)	7.86 (9.48)	10.31 (14.46)
Number of visits, pulmonology and allergy, mean (SD)	0.12 (1.18)	0.11 (1.25)	0.14 (1.07)
Number of visits, pulmonology, mean (SD)	2.33 (6.68)	2.01 (6.24)	2.83 (7.28)
Number of visits, ENT, mean (SD)	6.39 (8.27)	5.76 (6.24)	7.36 (10.57)
Frequency of visits, (visits/follow‐up, from first to last visit), mean (SD)	7.34 (15.01)	8.68 (18.2)	5.29 (7.6)
Frequency of visits, (visits/follow‐up, from first to end), mean (SD)	1.17 (1.48)	1.08 (1.36)	1.32 (1.63)
Time interval between visits (days), mean (SD)	207.43 (266.44)	213.66 (293.11)	197.91 (219.52)
Follow‐up time (years), mean (SD)	8.61 (3.5)	8.55 (3.5)	8.7 (3.5)
Allergy, *n* (%)	456 (33.6)	262 (32.03)	194 (35.99)
Asthma, *n* (%)	521 (38.39)	259 (31.66)	262 (48.61)
Chronic otitis media, *n* (%)	32 (2.36)	16 (1.96)	16 (2.97)
Diabetes, *n* (%)	152 (11.2)	81 (9.9)	71 (13.17)
Eosinophilia, *n* (%)	238 (17.54)	71 (8.68)	167 (30.98)
Immunodeficiency, *n* (%)	13 (0.96)	12 (1.47)	1 (0.19)
Immunodeficiency or its suspicion, *n* (%)	49 (3.61)	41 (5.01)	8 (1.48)
NERD, *n* (%)	119 (8.77)	28 (3.42)	91 (16.88)
Other chronic pulmonary diseases, *n* (%)	308 (22.7)	177 (21.64)	131 (24.3)
Tonsils disease, *n* (%)	66 (4.86)	50 (6.11)	16 (2.97)

Abbreviations: CRSsNP, chronic rhinosinusitis without nasal polyps; CRSwNP, chronic rhinosinusitis with nasal polyps; ENT, ear, nose, and throat diseases; NERD, non‐steroidal anti‐inflammatory drug exacerbated respiratory disease.

Comorbidities with the highest relative proportion were asthma (38%) and allergy (34%). These two comorbidities shared the highest relative proportion among patients with CRSwNP. The relative proportion of comorbid allergy among patients with CRSsNP and CRSwNP was 32% and 36%, respectively. The relative proportion of comorbid asthma among patients with CRSsNP and CRSwNP was 32% and 49%, respectively. The relative proportion of comorbid NERD among patients with CRSsNP and CRSwNP was 3.4% and 17%, respectively.

Other chronic pulmonary diseases existed in 23%, eosinophilia in 17%, diabetes in 11%, tonsils disease in 4.9%, immunodeficiency or its suspicion in 3.6%, and chronic otitis media in 2.4% of cases as comorbidity.

### 3.3 Overlapping diagnoses

We illustrated by Venn diagrams pediatric and adult patients with overlapping diagnoses of CRS and closely related comorbidities (Figure 1). The Venn diagrams show the absolute number of subjects having a diagnosis of interest. Notably, pediatric patients present a high overlap of allergy and asthma within CRS (*n* = 41).

### 3.4 The significant difference in comorbidities between children and adults

Odds ratios and 95% CI of logistic regression analysis evaluating the statistical difference in comorbidities between age groups are shown in Table 3. Comorbidities that significantly differed (*p*‐value <0.05) between pediatric and adult patients with CRS were [OR (95% CI)] allergy [0.39 (0.26–0.58)], chronic otitis media [0.1 (0.06–0.18)], and tonsils disease [0.19 (0.11–0.32)].

**TABLE 3:** Logistig regression analysis comparing comorbidities among pediatric and adult patients.

Variable	*N* (%)	OR (95% CI)
Allergy		**0.39 (0.26–0.58)**
Children	59 (56.73)	
Adults	456 (33.6)	
Asthma		0.7 (0.47–1.04)
Children	49 (47.12)	
Adults	521 (38.39)	
Chronic otitis media		**0.1 (0.06–0.18)**
Children	20 (19.23)	
Adults	32 (2.36)	
Diabetes		1.19 (0.6–2.33)
Children	10 (9.62)	
Adults	152 (11.2)	
Eosinophilia		1.49 (0.82–2.71)
Children	13 (12.5)	
Adults	238 (17.54)	
Immunodeficiency		1,211,117.2 (0–inf)
Children	0 (0)	
Adults	13 (0.96)	
Immunodeficiency or its suspicion		0.94 (0.33–2.65)
Children	4 (3.85)	
Adults	49 (3.61)	
NERD		1.9 (0.76–4.76)
Children	5 (4.81)	
Adults	119 (8.77)	
Other chronic pulmonary diseases		1.74 (0.99–3.05)
Children	15 (14.42)	
Adults	308 (22.7)	
Tonsils disease		**0.19 (0.11–0.32)**
Children	22 (21.15)	
Adults	66 (4.86)	

*Note*: Significant values (*p*‐value <0.05) are bolded.

Abbreviation: NERD, non‐steroidal anti‐inflammatory drug exacerbated respiratory disease.

### 3.5 Baseline endoscopic sinus surgery

The baseline endoscopic sinus surgeries performed for children are shown in Table S4. Baseline ESS was performed in 41% (*n* = 43) of the children. The relative proportion of pediatric CRSsNP and CRSwNP patients who underwent ESS was 40% (*n* = 36) and 47% (*n* = 7), respectively. The mean age ([min–max], SD) during baseline ESS was 15.8 ([6.1–28.1], 5.5) years among operated children. When observing the age of children in the group who had undergone baseline ESS, it was found that 26% (*n* = 11) of the patients were 12 years old or younger, 35% (*n* = 15) were between the ages of 13% and 17%, and 40%% (*n* = 17) were 18 years or older during the first surgery. The most common operation performed was the functional endoscopic opening of the maxillary antrum (DMB20). There were no children with periorbital cellulitis or abscess. Ethmoidectomy (DNB20) was divided into two categories, total and partial, based on the extent of the surgical treatment. In children, only one baseline ethmoidectomy was performed.

**TABLE 4:** Risk of revision after baseline endoscopic sinus surgery among adult patients using univariate Cox proportional hazard models.

Variable	*N* all	*N* events (%)	HR (95% CI)	*p*‐value
Age			2.9 (0.89–9.6)	0.078
Gender, female	367	55 (15)	1.1 (0.7–1.6)	0.78
Allergy	233	47 (20.2)	1.8 (1.2–2.7)	**0.0042**
Asthma	247	55 (22.3)	2.3 (1.5–3.4)	**<0.0001**
Baseline total ethmoidectomy	7	0 (0)	0.0000003 (0–inf)	0.99
Chronic otitis media	16	5 (31.2)	2.1 (0.84–5.1)	0.11
CRSwNP	257	59 (23)	2.6 (1.7–3.9)	**<0.0001**
Diabetes	59	9 (15.3)	1.1 (0.55–2.2)	0.8
Eosinophilia	147	32 (21.8)	1.9 (1.3–2.9)	**0.0029**
Immunodeficiency	2	1 (50)	3.1 (0.43–22)	0.26
Immunodeficiency or its suspicion	22	10 (45.5)	4.3 (2.2–8.3)	**<0.0001**
NERD	69	22 (31.9)	2.8 (1.7–4.5)	**<0.0001**
Number of any diseases ≥2	261	59 (22.6)	2.6 (1.7–3.9)	**<0.0001**
Obesity	55	8 (14.5)	1 (0.5–2.1)	0.95
Obstructive sleep apnea	59	13 (22)	1.7 (0.95–3.1)	0.071
Other chronic pulmonary diseases	137	26 (19)	1.5 (0.93–2.3)	0.1
Tonsils disease	27	2 (7.4)	0.49 (0.12–2)	0.32

*Note*: Age is reported at the time of the operation. *p*‐values <0.05 are bolded.

Abbreviations: CI, confidence interval; CRS, chronic rhinosinusitis; CRSwNP, chronic rhinosinusitis with nasal polyps; HR, hazard ratio; NERD, non‐steroidal anti‐inflammatory drug exacerbated respiratory disease.

The endoscopic sinus surgeries performed in adults are shown in Table S5. In adults, 46% (*n* = 627) underwent baseline ESS. The relative proportion of adult CRSsNP and CRSwNP patients who underwent ESS was 45% (*n* = 370) and 48% (*n* = 257), respectively. The mean age ([min–max], SD) during baseline ESS was 47.3 ([18.0–90.1], 15.0) years among operated adults. Functional endoscopic opening of the maxillary antrum is the most common operation performed. Two out of four adults who were reported to have periorbital cellulitis or abscess underwent functional endoscopic opening of the maxillary antrum. The other two did not undergo a surgical procedure. Ethmoidectomy was performed in 7.8% (*n* = 49) of the adults. Seven of these procedures were considered total ethmoidectomy.

Endonasal trephine of the maxillary antrum (DMB00) was the only operation that significantly (*p*‐value <0.05) differed between the children and the adults, and it was more commonly performed among the children.

### 3.6 Additional surgeries

In addition to ESS, additional tonsil or adenoid surgery or tympanostomy was performed for 22 children without, before, with, or after baseline ESS (see Table S1). The mean age ([min–max], SD) of the children who underwent additional surgery was 11.0 ([0.8–28.7], 8.2) years during the first operation. When observing the children who had additional surgery, 23% (*n* = 5) were 18 years or older during the operation. Adenoidectomy was performed on nine children with a mean age of (±SD) 8.1 (±4.3) years at the time of the operation. Three of these procedures were carried out independently of subsequent ESS (mean age ± SD 8.6 ± 7.8 years), two were performed before baseline ESS (mean age ± SD 10.7 ± 8.0 years) and four were performed together with ESS (mean age ± SD 13.4 ± 5.0 years). Functional endoscopic opening of the maxillary antrum is the most common procedure performed using adenoidectomy.

In adults, additional surgery was performed for 49 patients (see Table S2). The mean age ([min–max], SD) at the time of the first additional operation was 36.5 ([18.8–66.8], 17.0) years among the adults. Tonsillectomy was the most common additional surgery performed in adults. 76% (*n* = 31) of the adults who underwent tonsillectomy were operated before baseline ESS.

Additional surgery was performed significantly more frequently (OR 0.14, 95% CI 0.08–0.24) among children when compared to adults (see Table S3).

### 3.7 Factors affecting the relapse after baseline endoscopic sinus surgery

Fifteen % (*n* = 93) of adults who underwent baseline ESS required revision surgery. The variables entered in the Cox univariate regression model to evaluate factors contributing to relapse after baseline ESS in adult‐type CRS are shown in Table 4. Hazar ratios (95% CI) for female gender and age were 1.1 (0.72–1.6) and 2.9 (0.89–9.6) with *p*‐values of 0.78 and 0.078, respectively. The following conditions were associated with revision ESS (*p* < 0.05): allergy, asthma, CRSwNP, eosinophilia, immunodeficiency or its suspicion, NERD, and number of any diseases ≥2.

**FIGURE 2:**
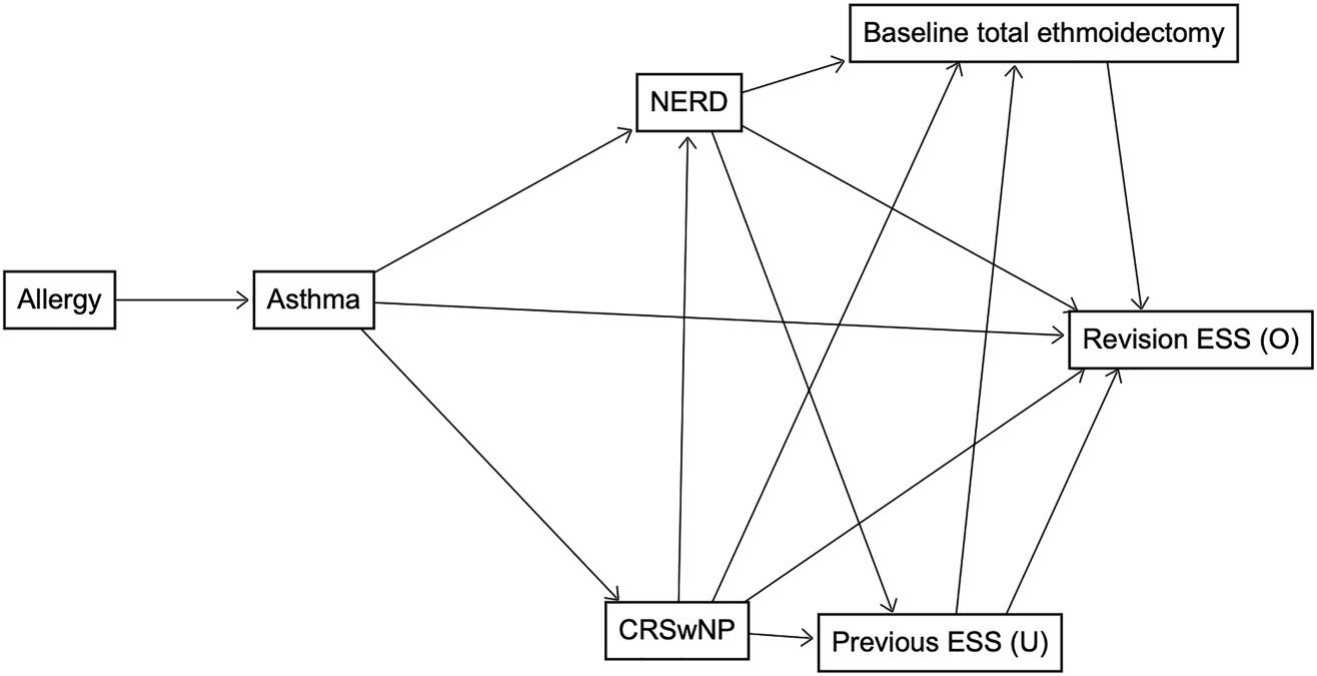
A directed acyclic graph of factors contributing to revision endoscopic sinus surgery. CRSwNP, chronic rhinosinusitis with nasal polyps. ESS, endoscopic sinus surgery; NERD, non‐steroidal anti‐inflammatory drug exacerbated respiratory disease; O, outcome; U, unobserved. Previous ESS indicates surgery underwent before baseline ESS.

Based on a DAG (see Figure 2), minimal sufficient adjustment sets were applied for asthma, CRSwNP and NERD to estimate the total effect of these exposure factors on revision ESS. Hazard ratios (95% CI) for asthma, CRSwNP, and NERD were 2.2 (1.5–3.4), 2.3 (1.5–3.6) and 1.6 (0.93–2.8), respectively.

## 4 DISCUSSION

This study was carried out to evaluate phenotypes, comorbidities, and surgical operations among pediatric and adult patients with CRS. Additionally, we evaluated risk factors that increase the frequency of revision ESS.

There is a limited understanding of PCRS and its associated comorbidities. In our study, allergy (53.2%) and asthma (41.3%) were the two most frequent comorbidities among PCRS patients. A previous study by Sedaghat et al.^25^ revealed that 26.9% of pediatric patients with CRS had concurrent allergic rhinitis (AR), while 18.1% had accompanying asthma diagnoses. Similar research conducted in Thailand^26^ and India^27^ reported that 53% of PCRS patients tested positive on skin prick tests. Consequently, our findings align more closely with these latter results than those of Sedaghat's study. Because of varying outcomes across studies, the exact role of AR in the development of CRS remains not fully elucidated.^1^ However, the comorbidity between asthma and rhinosinusitis has been noticed in both children and adults.^1^, ^3^ Currently, AR, CRS and asthma are viewed within the framework of the united airway theory, which treats upper and lower airways as a single functional entity sharing epidemiological and pathophysiological factors.^28^ As a result, CRS has been demonstrated to amplify the risk of developing asthma and vice versa.^1^, ^29^

In our study, 9.52% of PCRS patients had diabetes. Globally, Finland has the highest incidence of type I diabetes (T1D).^30^ Thus, our results might show a more significant relative proportion of CRS patients with T1D than other international studies. Moreover, an unexpectedly high relative proportion of pediatric diabetes in our study could be explained by more frequent visits with a physician as the patient has an underlying health condition, which could lead to better recognition of conditions that could otherwise stay undiagnosed. Thus, diabetic patients could be more easily diagnosed with general diseases such as rhinosinusitis. Nonetheless, the association between diabetes and upper respiratory tract infections (URTI) remains unclear. Previously, Muller et al.^31^ found no association between T1D and type II diabetes and UTRI. A study by Abu‐Ashour et al.^32^ did not differentiate upper and lower respiratory tract infections from each other; however, they found an association between diabetes and respiratory tract infections. Although it is acknowledged as a potential correlation, the proof reinforcing the association between URTI and diabetes appears less prominent than other types of infections, such as urinary tract infections.^31^, ^32^

We showed that chronic otitis media and tonsils disease affected one‐fifth of the pediatric group. The notable prevalence of these common diseases within our hospital cohort might be partially influenced by the higher occurrence of these conditions among children in the general populace.^33^ However, CRS, COM and tonsillitis share pathophysiological mechanisms such as biofilm bacteria.^34^, ^35^ Paranasal sinuses, middle ear and tonsils are close and contiguous structures, enabling the possible inflammation to spread and have action in all three complexes. Kim et al.^36^ showed that CRS was significantly associated with an increased incidence of COM during a 14‐year follow‐up period among patients of all ages and genders. The hypothesis that CRS is associated with COM has been recognized, but more knowledge is needed. Furthermore, limited literature supports the association between CRS and tonsillitis.

Allergy, asthma, and other chronic respiratory diseases were highly prevalent among adults with CRS. This is in line with previous studies showing that allergy,^1^ asthma,^1^ and other chronic pulmonary diseases^37^ are common comorbidities associated with CRS.

In the present study, the relative proportion of NERD was 3.97% in all pediatric patients and 20% among pediatric CRSwNP patients. In the adult group, the relative proportion of NERD was 8.05% among all patients and 16.7% among patients with CRSwNP. This corresponds to prior research indicating that the occurrence of NERD within the CRSwNP population is around 16%.^38^ However, the prevalence of NERD among pediatric CRSwNP patients has yet to be reported separately to our knowledge. Our results could indicate a similar prevalence of NERD in pediatric and adult CRSwNP patients. Nevertheless, no reliable conclusions can be made since the number of patients was small. More studies are needed to evaluate the prevalence of NERD in the pediatric CRSwNP population more accurately.

We showed eosinophilia as being the most prevalent in adults with CRSwNP. This aligns with prior research indicating that eosinophilia is more prevalent in polypotic patients than in non‐polypotic patients.^9^ In our study, pediatric CRSwNP patients had a lower relative proportion of eosinophilia than those without polyps. To our knowledge, the prevalence of eosinophilia among pediatric CRSwNP patients has yet to be reported. However, it has been proposed that immunological pathways leading to CRS would differ between adults and children, with PCRS being characterized by less tissue eosinophil infiltration than the adult form CRS.^8^, ^9^

Overall, we showed a high relative proportion of allergy, asthma, other chronic respiratory diseases, and eosinophilia among adult and pediatric patients treated for CRS in a hospital setting. We showed comorbidities that significantly differed (*p* < 0.05) between pediatric and adult patients were allergy, COM, and tonsils disease. One could argue that these results could also be observed in other populations. However, this could suggest that pediatric patients more often have underlying risk factors contributing to the development of PCRS as a heterogeneous disease. Thus, this reinforces the demand to identify which debated or possible exposures (such as allergy^1^ or COM^36^) play a role in the genesis of PCRS. Improved recognition and early PCRS intervention are needed to better understand the disease and prevent long‐term consequences.

We illustrated by Venn diagrams the overlap of CRS and closely related diseases. Both age groups showed a high overlap of allergy and asthma with CRS, but the overlap was more pronounced in children. There is a notable prevalence of overlap between NERD and CRSwNP in adults. It is worth noting that some asthma diagnoses may include allergic diseases, as asthma is often considered an allergic respiratory condition. This could contribute to the observed association between asthma and allergy.

We showed that 41% of the children and 46% of the adults underwent ESS, and there was an insignificant difference between the children and the adults who were surgically treated. Additionally, it was revealed that the relative proportion of pediatric and adult CRSwNP patients who underwent baseline ESS was 47% and 48%, respectively. In Finland, the reported nationwide average ESS rate is 0.71 per 1000 inhabitants,^39^ and a recent study showed that among 18,600 CRSwNP patients in Finland, ESS was performed for 47% of the patients.^40^ Therefore, our findings might reflect a comparable prevalence of ESS among CRS patients. However, it is important to acknowledge that our study was conducted in a single tertiary care hospital setting, where patients tend to present with more severe cases of rhinosinusitis.

Our study found maxillary antrostomy to be the predominant baseline procedure for pediatric and adult CRS patients. The procedure enhances sinus ventilation and drainage and enables comprehensive treatment access. Low complication rates reported in studies for both age groups might further support its common utilization.^41^, ^42^ Moreover, we showed that maxillary trephination (puncture) was the only operation that significantly differed between children and adults, with it being more commonly performed among children. Maxillary sinus trephination could be preferred in children due to its less invasive nature for diagnostic or therapeutic purposes compared with antrostomy surgery.

In addition to evaluating ESS, we also briefly assessed additional surgeries of the ear, nose, and pharynx: tonsillectomy, adenotonsillectomy, adenoidectomy, tympanostomy and balloon catheter sinuplasty. We detected that adenoidectomy was performed in 8.7% (*n* = 9) of the children in our cohort. The European Position Paper on Rhinosinusitis and Nasal Polyps guidelines suggest adenoidectomy as the first‐line procedure for treating CRS symptoms in younger patients.^1^ A study by Ramadan discovered that children above six had a better success rate after ESS than children younger than six.^43^ Our study reflects these recommendations and results as children who underwent adenoidectomy were younger than those who underwent ESS. In our study, we observed that 50% of children who underwent adenoidectomy subsequently required ESS, which aligns with current knowledge about the effectiveness of adenoidectomy for PCRS.^44^ Significant differences existed between the children and the adults who had undergone additional surgery. This is expected as minor operations are often considered for PCRS patients before opting for ESS due to the severity of the operation and the anatomy of paranasal sinuses, which continue to develop through childhood.

This study also evaluated factors associated with failure upon baseline ESS. We showed by regression analyses that the presence of allergy, asthma, NERD, eosinophilia, immunodeficiency or its suspicion, having two or more comorbid diseases, and nasal polyps increased the risk of revision ESS. This is in line with what has been shown in earlier research, as allergy, asthma, CRSwNP, and NERD have been shown to affect the result of ESS negatively.^1^, ^19^ The role of eosinophilia in polyp recurrence remains unclear, as the CRSwNP phenotype itself is often characterized by increased eosinophil levels.^1^ Moreover, immunodeficient CRS patients have been reported to experience equal benefits from ESS compared to immunocompetent patients.^45^ In our study, we observed a limited number of patients with immunodeficiency. Initially, immunodeficiency did not significantly impact on the requirement for revision ESS. A noteworthy shift only occurred when we incorporated suspicion of immunodeficiency into the variable. Thus, we did not include eosinophilia or immunodeficiency in our DAG because of a lack of evidence.

We did not detect an association between total ethmoidectomy and the need for revision of ESS. Caution should be taken when interpreting these results as the number of subjects who underwent total ethmoidectomy was small, which might cause an underpowered sample. Current knowledge suggests that more extensive surgery improves disease control in polypotic CRS.^46^ However, the evidence supporting extended surgeries is currently based on revision cases and not primary surgeries.^1^ A recent study showed that the outcome of total ethmoidectomy in CRSwNP patients with NERD is poorer than in non‐NERD patients.^20^ In our study, when examining the overall effect of asthma, CRSwNP and NERD on the need for revision of ESS, it was revealed that asthma and CRSwNP had the most significant impact. With higher costs and extended operating room time,^1^, ^47^ future studies should assess whether complete ethmoidectomy reduces overall healthcare costs in multimorbid adults.

## 5 STRENGTHS AND LIMITATIONS

The strengths of this study include a substantial and randomized sample of patients who sought outpatient care, as well as the utilization of text mining of EHR texts together with coded diagnoses. Our findings demonstrate that EHR information extraction effectively identifies patients with NERD and non‐respiratory coexisting conditions within rhinology patients.

A benefit of the study is that we used text mining technology, which made it possible to study a large random sample of hospital patients and provide new information on epidemiology, comorbidities and ESS rates.

Some limitations need discussion. In this register‐based study, some diseases might be missing in the EHR text. We studied a single‐center hospital cohort that might differ somewhat from a population‐based situation. Moreover, due to a university hospital cohort, only the most severe cases of rhinosinusitis might be included, as patients with more lenient symptoms could seek help from the private sector. On the other hand, about ¼ or 1/5 of the Finnish population lives in the Hospital District of Helsinki and Uusimaa, reflecting a significant part of the population. We acknowledge that due to this hospital cohort setup, the study population lacked a control group.

Cystic fibrosis and primary ciliary dyskinesia are rare autosomal disorders but are often seen together with PCRS.^48^, ^49^ It has been shown that nasal polyps might affect up to 86% of pediatric CF patients.^48^ Unfortunately, our cohort had no CF or PCD patients. Finland has a particularly low incidence of CF.^50^ As our study was conducted on a random sample of rhinosinusitis patients, the absence of PCRS patients diagnosed with either CF or PCD may be due to chance or the rarity of these diseases.

We were unable to identify the number of rhinosinusitis patients with odontogenic problems. Research indicates that approximately 10% of adult CRS may be linked to potential odontogenic causes.^51^ These causes include maxillary root infections or oro‐antral fistulas resulting from tooth extractions. Unfortunately, dental information is frequently underrepresented in patient records, and radiological reports may not be available. Moreover, the absence of a distinct ICD‐10 code for odontogenic sinusitis complicates the identification of CRS with odontogenic origins in register‐based studies.

We recognize that suspicion of immunodeficiency differs from a confirmed diagnosis of immunodeficiency. However, suspicion of immunodeficiency may indirectly indicate a similar situation characterized by poor CRS control, prompting physicians to consider this rare comorbidity.

The study's retrospective design, focus on a specific hospital population, and the possibility of incomplete data extraction due to coding limitations introduced certain constraints. Information on visits to general practitioners, occupational healthcare, or the private sector was not available. The study also lacked a control group and data on symptom scores, medications, polyp scores, and Lund‐Mackay scores. Additionally, the role of sinus surgery has been analyzed elsewhere. The relative proportion of diagnoses observed in the study may not completely align with their prevalence, potentially accounting for variations compared with findings from general population studies. Hence, the results require verification in other population groups, and examining various healthcare sectors within Finland would yield important insights into the comprehensive impact of chronic and recurrent acute rhinosinusitis.

## 6 CONCLUSIONS

Both pediatric and adult rhinosinusitis patients exhibited a notable prevalence of comorbidities such as allergy and asthma. However, in children, the prevalence of allergy, both individually and in conjunction with asthma, was significantly higher. Conditions such as COM and tonsils disease were also more prevalent among children. Future studies should evaluate the possible role of these exposures in the development of PCRS. ESS was equally prevalent in children and adults with CRS treated in a tertiary care setting. Notably, children underwent additional operations of the ear, nose and pharynx more frequently. Risk of revision ESS was associated, for example, with allergy, asthma, CRSwNP, NERD, and multiple comorbid diseases. Thus, the effectiveness of ESS in multimorbid adults should be conducted on a personalized basis.

## AUTHOR CONTRIBUTIONS

All authors participated in the planning and conception of the study and the analytical strategy. Aada Murtomäki, Mikko Nuutinen and Sanna Toppila‐Salmi performed the data analyses and wrote the manuscript. All authors have assisted in data management, analyses and critical review of the manuscript.

## CONFLICT OF INTEREST STATEMENT

STS reports consultancies for ALK‐Abelló, AstraZeneca, Clario, ERT, GlaxoSmithKline, Novartis, Sanofi Pharma, OrionPharma, Roche Products, and a grant from GlaxoSmithKline and Sanofi. All are outside the submitted work. All other authors declare no conflicts of interest.

## CONSENT FOR PUBLICATION

Not applicable.

## Supporting information

Table S1

Table S2

Table S3

Table S4

Table S5

## Data Availability

Due to Finnish data protection legislation and confidential health‐related data, the datasets produced and/or examined during this study are not accessible to the general public. They can solely be managed by designated individuals within the study group for specific research objectives. The datasets analyzed during the current study are available from the corresponding author upon reasonable request. Data use permissions can be applied from the competent authorities.
