# Supplementary material for: Comorbidities of chronic rhinosinusitis in children and adults
Source: Clin Transl Allergy. 2024 Apr 24;14(4):e12354. doi: 10.1002/clt2.12354 (PMC11043011; doi:10.1002/clt2.12354)
Supplement: Supplementary file 1 — Table S1 [file CLT2-14-e12354-s001.docx]

| **Operation time** | **Any additional operation** | **DCA20** | **EMB10** | **EMB20** | **EMB30** | **ZXC87** |
| --- | --- | --- | --- | --- | --- | --- |
| Operated at any time | 22 (100) | 4 (100) | 6 (100) | 3 (100) | 9 (100) | 0 (NaN) |
| No BESS | 7 (31.82) | 2 (50) | 1 (16.67) | 1 (33.33) | 3 (33.33) | 0 (NaN) |
| Before BESS | 7 (31.82) | 2 (50) | 3 (50) | 0 (0) | 2 (22.22) | 0 (NaN) |
| Together with BESS | 7 (31.82) | 0 (0) | 1 (16.67) | 2 (66.67) | 4 (44.44) | 0 (NaN) |
| After BESS | 1 (4.55) | 0 (0) | 1 (16.67) | 0 (0) | 0 (0) | 0 (NaN) |

**Supplementary Table 1**: Additional operations of ear, nose and pharynx among children. BESS = baseline endoscopic sinus surgery. DCA20 = tympanostomy. EMB10 = tonsillectomy. EMB20 = adenotonsillectomy. EMB30 = adenoidectomy. ZXC87 = balloon catheter sinuplasty
