# Supplementary material for: Comorbidities of chronic rhinosinusitis in children and adults
Source: Clin Transl Allergy. 2024 Apr 24;14(4):e12354. doi: 10.1002/clt2.12354 (PMC11043011; doi:10.1002/clt2.12354)
Supplement: Supplementary file 2 — Table S2 [file CLT2-14-e12354-s002.docx]

| **Operation time** | **Any additional operation** | **DCA20** | **EMB10** | **EMB20** | **EMB30** | **ZXC87** |
| --- | --- | --- | --- | --- | --- | --- |
| Operated at any time | 49 (100) | 6 (100) | 41 (100) | 0 (NaN) | 2 (100) | 0 (NaN) |
| No BESS | 24 (48.98) | 2 (33.33) | 22 (53.66) | 0 (NaN) | 0 (0) | 0 (NaN) |
| Before BESS | 10 (20.41) | 1 (16.67) | 9 (21.95) | 0 (NaN) | 0 (0) | 0 (NaN) |
| Together with BESS | 4 (8.16) | 2 (33.33) | 0 (0) | 0 (NaN) | 2 (100) | 0 (NaN) |
| After BESS | 11 (22.45) | 1 (16.67) | 10 (24.39) | 0 (NaN) | 0 (0) | 0 (NaN) |

**Supplementary Table 2**: Additional operations of ear nose and pharynx among adults. BESS = baseline endoscopic sinus surgery. DCA20 = tympanostomy. EMB10 = tonsillectomy. EMB20 = adenotonsillectomy. EMB30 = adenoidectomy. ZXC87 = balloon catheter sinuplasty
