# Supplementary material for: Comorbidities of chronic rhinosinusitis in children and adults
Source: Clin Transl Allergy. 2024 Apr 24;14(4):e12354. doi: 10.1002/clt2.12354 (PMC11043011; doi:10.1002/clt2.12354)
Supplement: Supplementary file 3 — Table S3 [file CLT2-14-e12354-s003.docx]

| **Operated at any time** | **N (%)** | **OR (95% CI)** | **No BESS** | **N (%)** | **OR (95% CI)** | **Before BESS** | **N (%)** | **OR (95% CI)** | **Together with BESS** | **N (%)** | **OR (95% CI)** | **After BESS** | **N (%)** | **OR (95% CI)** |
| --- | --- | --- | --- | --- | --- | --- | --- | --- | --- | --- | --- | --- | --- | --- |
| **Any additional operation** |  | 0.14 (0.08-0.24) | **Any additional operation** |  | 2.06 (0.71-5.92) | **Any additional operation** |  | 0.55 (0.18-1.71) | **Any additional operation** |  | 0.19 (0.05-0.74) | **Any additional operation** |  | 6.08 (0.73-50.41) |
| Children | 22 (21.15) |  | Children | 7 (6.73) |  | Children | 7 (6.73) |  | Children | 7 (6.73) |  | Children | 1 (0.96) |  |
| Adults | 49 (3.61) |  | Adults | 24 (1.77) |  | Adults | 10 (0.74) |  | Adults | 4 (0.29) |  | Adults | 11 (0.81) |  |
| **DCA20** |  | 0.11 (0.03-0.4) | **DCA20** |  | 0.43 (0.06-3.24) | **DCA20** |  | 0.21 (0.02-2.43) | **DCA20** |  | 7326168.88 (0-Inf) | **DCA20** |  | 2638997.56 (0-Inf) |
| Children | 4 (3.85) |  | Children | 2 (1.92) |  | Children | 2 (1.92) |  | Children | 0 (0) |  | Children | 0 (0) |  |
| Adults | 6 (0.44) |  | Adults | 2 (0.15) |  | Adults | 1 (0.07) |  | Adults | 2 (0.15) |  | Adults | 1 (0.07) |  |
| **EMB10** |  | 0.51 (0.21-1.23) | **EMB10** |  | 17.11 (2.13-137.42) | **EMB10** |  | 1.42 (0.35-5.87) | **EMB10** |  | 0 (0-Inf) | **EMB10** |  | 5.38 (0.64-44.98) |
| Children | 6 (5.77) |  | Children | 1 (0.96) |  | Children | 3 (2.88) |  | Children | 1 (0.96) |  | Children | 1 (0.96) |  |
| Adults | 41 (3.02) |  | Adults | 22 (1.62) |  | Adults | 9 (0.66) |  | Adults | 0 (0) |  | Adults | 10 (0.74) |  |
| **EMB20** |  | 0 (0-Inf) | **EMB20** |  | 0 (0-Inf) | **EMB20** |  | NA (NA-NA) | **EMB20** |  | 0 (0-Inf) | **EMB20** |  | NA (NA-NA) |
| Children | 3 (2.88) |  | Children | 1 (0.96) |  | Children | 0 (0) |  | Children | 2 (1.92) |  | Children | 0 (0) |  |
| Adults | 0 (0) |  | Adults | 0 (0) |  | Adults | 0 (0) |  | Adults | 0 (0) |  | Adults | 0 (0) |  |
| **EMB30** |  | 0.02 (0-0.07) | **EMB30** |  | 0 (0-Inf) | **EMB30** |  | 0 (0-Inf) | **EMB30** |  | 0.19 (0.03-1.14) | **EMB30** |  | NA (NA-NA) |
| Children | 9 (8.65) |  | Children | 3 (2.88) |  | Children | 2 (1.92) |  | Children | 4 (3.85) |  | Children | 0 (0) |  |
| Adults | 2 (0.15) |  | Adults | 0 (0) |  | Adults | 0 (0) |  | Adults | 2 (0.15) |  | Adults | 0 (0) |  |
| **ZXC87** |  | NA (NA-NA) | **ZXC87** |  | NA (NA-NA) | **ZXC87** |  | NA (NA-NA) | **ZXC87** |  | NA (NA-NA) | **ZXC87** |  | NA (NA-NA) |
| Children | 0 (0) |  | Children | 0 (0) |  | Children | 0 (0) |  | Children | 0 (0) |  | Children | 0 (0) |  |
| Adults | 0 (0) |  | Adults | 0 (0) |  | Adults | 0 (0) |  | Adults | 0 (0) |  | Adults | 0 (0) |  |

**Supplementary Table 3**: Logistic regression analysis comparing additional surgeries of ear, nose and pharynx among pediatric and adult patients. BESS = baseline endoscopic sinus surgery. DCA20 = tympanostomy. EMB10 = tonsillectomy. EMB20 = adenotonsillectomy. EMB30 = adenoidectomy. ZXC87 = balloon catheter sinuplasty
