# Supplementary material for: Comorbidities of chronic rhinosinusitis in children and adults
Source: Clin Transl Allergy. 2024 Apr 24;14(4):e12354. doi: 10.1002/clt2.12354 (PMC11043011; doi:10.1002/clt2.12354)
Supplement: Supplementary file 4 — Table S4 [file CLT2-14-e12354-s005.docx]

*Supplementary Table 4: Baseline endoscopic sinus surgery among children.*

| **Variable** | **Any code** | **DHB20** | **DMB00** | **DMB20** | **DNB20** | **DNB20 total** | **DNB20 partial** | **DNB30** | **DPA20** | **DPA25** | **DPA30** |
| --- | --- | --- | --- | --- | --- | --- | --- | --- | --- | --- | --- |
| All operated, n (%) | 43 (100) | 3 (6.98) | 9 (20.93) | 28 (65.12) | 1 (2.33) | 0 (0) | 1 (2.33) | 1 (2.33) | 0 (0) | 0 (0) | 1 (2.33) |
| Allergy, n (%) | 20 (33.9) | 1 (1.69) | 6 (10.17) | 12 (20.34) | 0 (0) | 0 (0) | 0 (0) | 1 (1.69) | 0 (0) | 0 (0) | 0 (0) |
| Asthma, n (%) | 17 (34.69) | 1 (2.04) | 4 (8.16) | 11 (22.45) | 0 (0) | 0 (0) | 0 (0) | 1 (2.04) | 0 (0) | 0 (0) | 0 (0) |
| Chronic otitis media, n (%) | 10 (50) | 0 (0) | 5 (25) | 5 (25) | 0 (0) | 0 (0) | 0 (0) | 0 (0) | 0 (0) | 0 (0) | 0 (0) |
| Diabetes, n (%) | 4 (40) | 0 (0) | 2 (20) | 2 (20) | 0 (0) | 0 (0) | 0 (0) | 0 (0) | 0 (0) | 0 (0) | 0 (0) |
| Eosinophilia, n (%) | 5 (38.46) | 0 (0) | 1 (7.69) | 3 (23.08) | 0 (0) | 0 (0) | 0 (0) | 1 (7.69) | 0 (0) | 0 (0) | 0 (0) |
| Immunodeficiency, n (%) | 0 (NaN) | 0 (NaN) | 0 (NaN) | 0 (NaN) | 0 (NaN) | 0 (NaN) | 0 (NaN) | 0 (NaN) | 0 (NaN) | 0 (NaN) | 0 (NaN) |
| Immunodeficiency or its suspicion, n (%) | 2 (50) | 0 (0) | 1 (25) | 0 (0) | 0 (0) | 0 (0) | 0 (0) | 1 (25) | 0 (0) | 0 (0) | 0 (0) |
| NERD, n (%) | 1 (20) | 1 (20) | 0 (0) | 0 (0) | 0 (0) | 0 (0) | 0 (0) | 0 (0) | 0 (0) | 0 (0) | 0 (0) |
| Other chronic pulmonary diseases, n (%) | 8 (53.33) | 1 (6.67) | 4 (26.67) | 3 (20) | 0 (0) | 0 (0) | 0 (0) | 0 (0) | 0 (0) | 0 (0) | 0 (0) |
| Tonsils disease, n (%) | 14 (63.64) | 0 (0) | 7 (31.82) | 6 (27.27) | 0 (0) | 0 (0) | 0 (0) | 0 (0) | 0 (0) | 0 (0) | 1 (4.55) |

**Info:** The child population was defined as individuals who were under 18 years old at the time of their first visit

**Abbreviations:** DHB20 = Polypectomy of internal nose. DMB00 = Endonasal trephine of maxillary antrum. DMB20 = Functional endoscopic opening of maxillary antrum. DHB20 = Polypectomy of internal nose. DNB30 = Excision of lesion of ethmoidal sinus. DPA20 = Trephination of frontal sinus. DPA25 = Trephination of frontal sinus through nose. DPA30 = Sphenotomy. NERD = non-steroidal anti-inflammatory drug exacerbated respiratory disease.
