# Supplementary material for: Comorbidities of chronic rhinosinusitis in children and adults
Source: Clin Transl Allergy. 2024 Apr 24;14(4):e12354. doi: 10.1002/clt2.12354 (PMC11043011; doi:10.1002/clt2.12354)
Supplement: Supplementary file 5 — Table S5 [file CLT2-14-e12354-s004.docx]

*Supplementary Table 5: Baseline endoscopic sinus surgery among adults.*

| **Variable** | **Any code** | **DHB20** | **DMB00** | **DMB20** | **DNB20** | **DNB20 total** | **DNB20 partial** | **DNB30** | **DPA20** | **DPA25** | **DPA30** |
| --- | --- | --- | --- | --- | --- | --- | --- | --- | --- | --- | --- |
| All operated, n (%) | 627 (100) | 85 (13.56) | 5 (0.8) | 455 (72.57) | 49 (7.81) | 7 (1.12) | 42 (6.7) | 7 (1.12) | 5 (0.8) | 11 (1.75) | 10 (1.59) |
| Allergy, n (%) | 233 (51.1) | 27 (5.92) | 0 (0) | 173 (37.94) | 27 (5.92) | 4 (0.88) | 23 (5.04) | 1 (0.22) | 2 (0.44) | 2 (0.44) | 1 (0.22) |
| Asthma, n (%) | 247 (47.41) | 46 (8.83) | 1 (0.19) | 169 (32.44) | 27 (5.18) | 3 (0.58) | 24 (4.61) | 0 (0) | 1 (0.19) | 3 (0.58) | 0 (0) |
| Chronic otitis media, n (%) | 16 (50) | 3 (9.38) | 0 (0) | 11 (34.38) | 2 (6.25) | 0 (0) | 2 (6.25) | 0 (0) | 0 (0) | 0 (0) | 0 (0) |
| Diabetes, n (%) | 59 (38.82) | 7 (4.61) | 0 (0) | 48 (31.58) | 3 (1.97) | 1 (0.66) | 2 (1.32) | 0 (0) | 0 (0) | 0 (0) | 1 (0.66) |
| Eosinophilia, n (%) | 147 (61.76) | 26 (10.92) | 1 (0.42) | 95 (39.92) | 23 (9.66) | 4 (1.68) | 19 (7.98) | 0 (0) | 1 (0.42) | 0 (0) | 1 (0.42) |
| Immunodeficiency, n (%) | 2 (15.38) | 0 (0) | 0 (0) | 2 (15.38) | 0 (0) | 0 (0) | 0 (0) | 0 (0) | 0 (0) | 0 (0) | 0 (0) |
| Immunodeficiency or its suspicion, n (%) | 22 (44.9) | 0 (0) | 0 (0) | 19 (38.78) | 1 (2.04) | 0 (0) | 1 (2.04) | 0 (0) | 0 (0) | 0 (0) | 2 (4.08) |
| NERD, n (%) | 69 (57.98) | 22 (18.49) | 1 (0.84) | 33 (27.73) | 9 (7.56) | 1 (0.84) | 8 (6.72) | 0 (0) | 1 (0.84) | 2 (1.68) | 1 (0.84) |
| Other chronic pulmonary diseases, n (%) | 137 (44.48) | 17 (5.52) | 1 (0.32) | 102 (33.12) | 15 (4.87) | 4 (1.3) | 11 (3.57) | 0 (0) | 0 (0) | 0 (0) | 2 (0.65) |
| Tonsils disease, n (%) | 27 (40.91) | 1 (1.52) | 0 (0) | 26 (39.39) | 0 (0) | 0 (0) | 0 (0) | 0 (0) | 0 (0) | 0 (0) | 0 (0) |

**Info:** The adult population was defined as individuals who were 18 years or older at the time of their first visit.

**Abbreviations**: Baseline endoscopic sinus surgery among adults. DHB20 = Polypectomy of internal nose. DMB00 = Endonasal trephine of maxillary antrum. DMB20 = Functional endoscopic opening of maxillary antrum. DHB20 = Polypectomy of internal nose. DNB30 = Excision of lesion of ethmoidal sinus. DPA20 = Trephination of frontal sinus. DPA25 = Trephination of frontal sinus through nose. DPA30 = Sphenotomy. NERD = non-steroidal anti-inflammatory drug exacerbated respiratory disease.
